# Persistence and continuous evolution of the human respiratory syncytial virus in northern Taiwan for two decades

**DOI:** 10.1038/s41598-019-41332-9

**Published:** 2019-03-18

**Authors:** Hsin Chi, Kuang-Liang Hsiao, Li-Chuan Weng, Chang-Pan Liu, Hsin-Fu Liu

**Affiliations:** 1 Department of Medicine, MacKay Medicine College, New Taipei, Taiwan; 2 Department of Pediatrics, MacKay Children’s Hospital and MacKay Memorial Hospital, Taipei, Taiwan; 3 Department of Medical Research, MacKay Memorial Hospital, Taipei, Taiwan; 4 Division of Infectious Diseases, Department of Internal Medicine, MacKay Memorial Hospital, Taipei, Taiwan; 5 Institute of Bioscience and Biotechnology, National Taiwan Ocean University, Keelung, Taiwan

## Abstract

The study aimed to characterize the molecular epidemiology, phylogenetic relationship, and population dynamics of the G protein gene in clinical respiratory syncytial virus (RSV) strains isolated from northern Taiwan. We analyzed a total of 160 and 116 G protein gene sequences of RSV-A and RSV-B representative strains, respectively, from 804 clinical viral stocks collected between July 2000 and June 2016. Population dynamic patterns of the RSV G protein gene were analyzed using Bayesian inference through the Markov chain Monte Carlo framework. A phylogenetic analysis revealed that RSV-A from Taiwan could be categorized into GA2, GA5, and GA7 lineages. GA2 of RSV-A could be further divided into NA1, NA2, NA4, and ON1 clades. These RSV-A lineages has been replaced over time, whereas RSV-B strains from Taiwan continually evolved from a single lineage with significant time-dependent waves. Four putative positive selection sites were observed in both RSV-A and RSV-B. The Bayesian skyline plot revealed that the local population dynamics of RSV were associated with lineage displacement events. Both circulating subtypes and population dynamics represented a unique local pattern. Our results affirm the necessity of continuing molecular surveillance of RSV to attain a more comprehensive understanding of epidemics.

## Introduction

Respiratory syncytial virus (RSV) infection is the leading cause of acute respiratory tract disease in children younger than 5 years^1^. RSV is an enveloped, nonsegmented, negative-sense RNA virus of family *Pneumoviridae*^2^. There is a single RSV serotype and two major antigenic subgroups (A and B), based on monoclonal antibody detection of the G protein together with the F protein^3^. Mature G protein comprise 3 regions: a cytoplasmic tail (amino acids [AAs] 1–38), a transmembrane domain (AA 38–66), and an ectodomain (AA 66–298)^4^. ON1, an RSV-A genotype with a characteristic 72-nucleotide duplication in the G protein gene, was first identified in Ontario, Canada in 2010 and quickly spread globally^5,6^. The specific RSV-B genotype, containing a 60-nucleotide duplication in the G protein gene, was termed “BA” for Buenos Aires in the late 1990s and has since been isolated from different graphic regions globally^7^.

The G protein is to facilitate the attachment of viruses to host cells, is also plays an immunomodulatory role during RSV infection^8,9^. Evidence suggests that positive selection accounts for the amino acid variability in G protein^10^. Genetic mutations in glycosylation sites of G protein were discovered in RSV and may play a role in immune escape^11^. Although the genetic features of RSV have been investigated in many countries, the evolution of RSV G protein and its population dynamics in Taiwan remains unclear in Taiwan. In this study, we isolated 160 and 116 G protein gene sequences of RSV-A and RSV-B representative strains, respectively, from 804 clinical samples and analyzed their phylogenetic relationship and population dynamics.

## Results

### Proportion of RSV genotypes

The proportion of RSV genotypes were defined by PCR product that showed correct size by agarose gel electrophoresis. Out of the 804 samples tested, 439 (55%) were RSV-A, 234 (29%) were RSV-B, 52 (6%) were RSV-A and RSV-B, and 79 (10%) were failed to detected by PCR (Table 1).

**Table 1:** Summaries of representative strains and their collection date.

Year	00*	01	02	03	04	05	06	07	08	09	10	11	12	13	14	15	16**	Total
Sapmple tested***																		
	11	9	25	29	30	28	30	46	60	65	76	65	60	57	58	72	83	804
**PCR typing**																		
RSV-A	4	4	19	24	23	12	28	20	37	27	53	50	31	26	24	36	21	439
(%)	36	44	76	83	77	43	93	43	62	42	70	77	52	46	41	50	25	55
RSV-B	6	4	5	4	7	16	2	14	18	28	13	6	10	12	19	19	51	234
(%)	55	44	20	14	23	57	7	30	30	43	17	9	17	21	33	26	61	29
A&B	1	1	1	1	0	0	0	8	5	7	5	3	3	0	1	9	7	52
(%)	9	11	4	3	0	0	0	17	8	11	7	5	5	0	2	13	8	6
Untypable	0	0	0	0	0	0	0	4	0	3	5	6	16	19	14	8	4	79
(%)	0	0	0	0	0	0	0	9	0	5	7	9	27	33	24	11	5	10
**Sequencing completed**																		
RSV-A	2	3	14	10	7	9	9	8	12	12	27	21	14	22	14	17	4	205
RSV-B	7	5	4	5	7	8	2	4	10	8	9	7	9	9	12	13	14	133
**Unique sequences**																		
RSV-A	2	3	11	8	6	5	7	7	5	8	19	21	14	18	9	15	2	160
RSV-B	5	5	4	5	6	8	1	4	9	4	9	6	7	9	11	13	10	116

^*^Samples were collected from July.

**Samples were collected until the end of June.

***Clinical samples were randomly collected, the sample size did not reflect to the number of hospitalized patients.

Unique sequences from this study were deposited in the GenBank database and assigned with accession numbers MF496651-MF496377 and MH045601.

### Phylogenetic analysis

**Figure 1:**
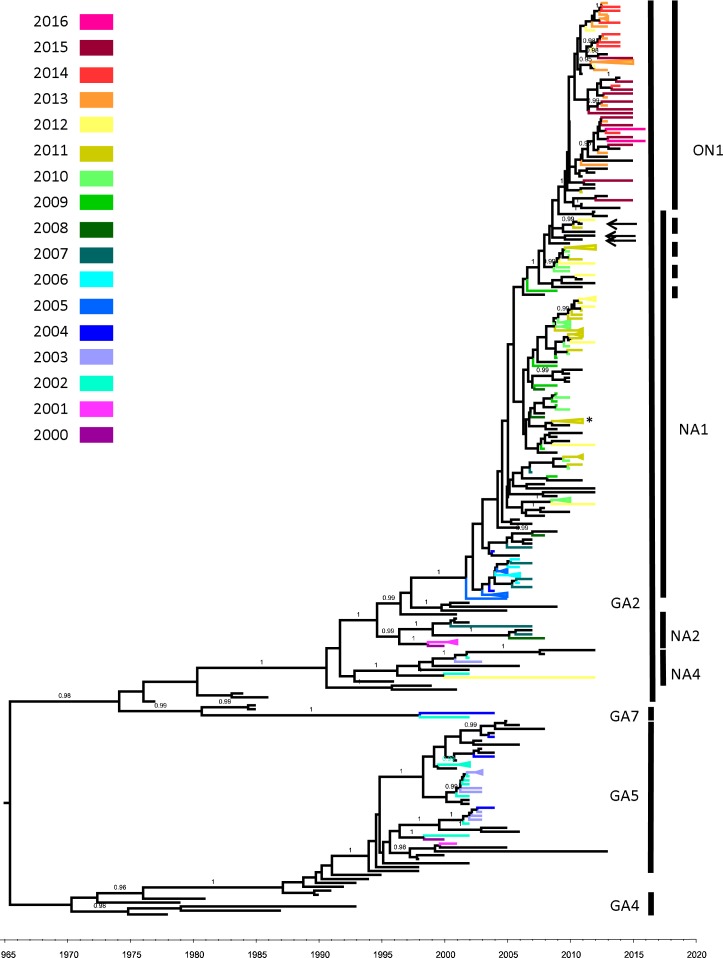
Unrooted Bayesian-inferred maximum clade credibility tree of the RSV-A G protein gene. Posterior probabilities are labeled on major branches. Branches with a posterior probability equal or greater than 0.95 were considered to be strongly supported. Isolates from this study were labeled on their branches using colors correlating to their collection date (year). The X axis indicates the timescale (year). Vertical bar indicates subtypes of RSV-A. The GA2 subtypes were further divided into NA1, NA2, NA4, and ON1 clades. The overlap between NA1 and ON1 indicated an intermediated region that included both with and without 72-bp duplicated strains. Arrows indicated the duplicated strains (KX765894, KX765936, and KX765960) that clustered with non-duplicated strains. Details regarding the reference strains involved in the Bayesian trees are presented in Supplementary Fig. S1. *Strain that isolated from putative co-infection sample (Detail regarding the related subtree is presented in Supplementary Fig. S2).

A total of 160 RSV-A and 116 RSV-B representative strains from Taiwan (Table 1) were obtained and phylogenetically analyzed after removing the identical sequences. The nucleotide sequence involved in the phylogenetic analysis corresponded to 298–891 nt (100–297 a.a.) and 271–873 nt (91–291 a.a.) of G gene coding region from the RSV-A reference strain M11486 and RSV-B reference strain AF013254, respectively. Based on the G gene Bayesian tree, all known RSV-A subtypes were statistically supported by posterior probability (pp) of >0.95. Of the 160 RSV-A strains, 20 belonged to GA5 (collected from 2000–2004), 2 belonged to GA7 (2002 and 2004), and 138 belonged to GA2 (2000–2016). Of the GA2 strains, 4 belonged to NA4 (2002–2003 and 2012), 6 belonged to NA2 (2000–2001 and 2007–2008), 80 belonged to NA1 (2004–2012), and 48 belonged to ON1 (2011–2016) (Fig. 1).

**Figure 2:**
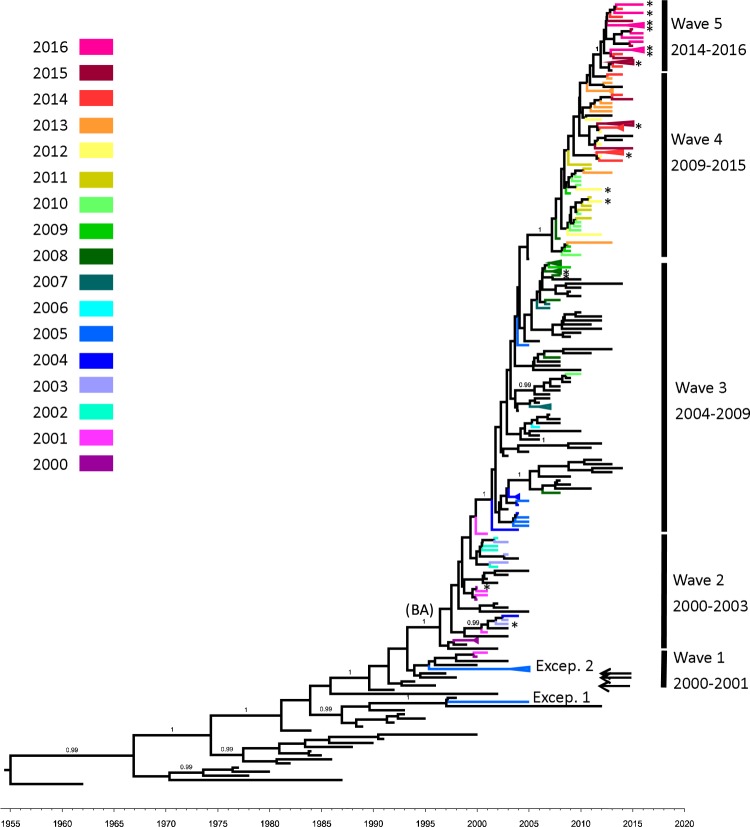
Unrooted Bayesian-inferred maximum clade credibility tree of the RSV-B G protein gene. Posterior probabilities are labeled on major branches. Branches with a posterior probability equal or greater than 0.95 were considered to be strongly supported. Isolates from this study were labeled on their branches using colors correlating to their collection date (year). The X axis indicates the timescale (year). Vertical bar indicates the 5 waves revealed from our data; each wave likely emerged from a surviving strain from the previous wave. The numbers below each wave refer to the sample collection dates for each specific wave, excluding reference strains. Arrows indicated the duplicated strains (KU316158, KU316172, and KU316105) that clustered with non-duplicated strains. Details regarding the reference strains involved in the Bayesian trees are presented in Supplementary Fig. S1. *Strains that isolated from putative co-infection samples (Details regarding the related subtree are presented in Supplementary Fig. S2).

Although traditional RSV-B subtypes were not well supported on the RSV-B Bayesian tree, RSV-B strains revealed 5 continuously evolving lineages with high statistical support (Fig. 2). During 2000–2001, waves 1 and 2 co-circulated in Taiwan, with 2 exceptions that were isolated in 2005 (excep.1 and except. 2 of Fig. 2). Wave 2, the initial wave of BA subtypes in Taiwan, became predominant after 2002. The RSV-B Taiwan strains isolated during 2004–2009, 2009–2015, and 2014–2016 were included in waves 3, 4, and 5 of the BA strains respectively.

### Evolutionary rates

The mean rate of molecular evolution for RSV-A and RSV-B was estimated to be approximately 2.26 × 10^−3^ and 2.87 × 10^−3^ substitutions/site/year respectively (see Table 2 for details). The evolutionary rate of the RSV G gene was approximately 2.5-fold faster than that of the F gene^12^.

**Table 2:** Evolution rates of RSV G protein gene.

RSV-A	Mean rate	Relative rates of each codon position		
		1^st^	2^nd^	3rd
substitution rate	2.26 × 10^−3^	0.69	1.03	1.28
95%HPD upper	2.61 × 10^−3^	0.80	1.19	1.43
95%HPD lower	1.93 × 10^−3^	0.57	0.87	1.12
RSV-B		1^st^	2^nd^	3rd
substitution rate	2.87 × 10^−3^	0.75	0.79	1.46
95%HPD upper	3.30 × 10^−3^	0.89	0.92	1.62
95%HPD lower	2.46 × 10^−3^	0.61	0.66	1.31

### Population dynamic analysis

**Figure 3:**
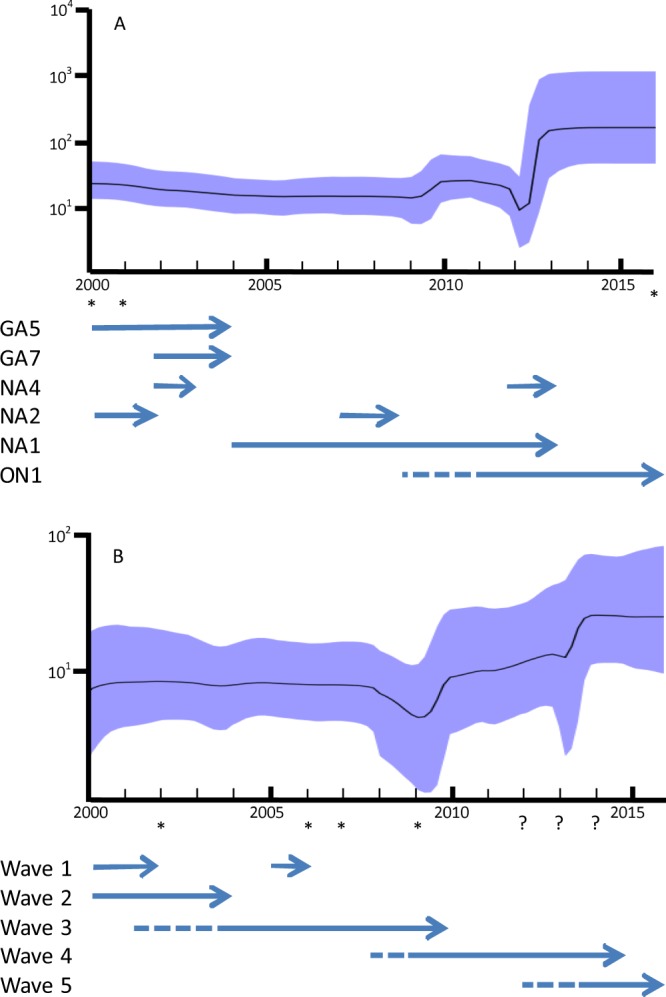
Population dynamics of the RSV determined using the Bayesian skyline plot method. Skyline plots represent the population dynamics of RSV in Taiwan. The Y-axis indicates the effective population size, and the X-axis indicates the time (year). The bold line indicates the estimated mean of the effective population size, and the colored margin indicates the range of 95% HPD. 2a: Skyline plots of RSV-A in Taiwan. The horizontal arrows indicated the subtype replacement over time. The dash line indicated the estimated tMRCA of ON1 subtype. 2b: Skyline plots of RSV-B in Taiwan. The horizontal arrows indicated the wave replacement over time. The dash line indicated the estimated tMRCA of waves 3, 4 and 5. *Sample size in these years was less than 5 and may not be able to represent their diversity. Question marks indicated the years that have more than 25% samples were untypable and the effective population size may be underestimated.

Bayesian skyline plots describing the population dynamics of RSV-A and RSV-B in Taiwan are presented in Fig. 3. The results for RSV-A revealed a steady effective population until 2000, a slow decline in its genetic variation until 2005, a rapid recovery to the basal level in 2009–2010, and then a sharp decrease in 2012 (Fig. 3A). By contrast, the effective population of RSV-B remained steady until 2008, followed by a sudden decrease, and then recovery to the basal level in 2010 (Fig. 3B). Both RSV-A and RSV-B subsequently demonstrated a significant increase until 2013.

### Selection pressure

To estimate the selection pressure that may emerge after recent coalescent events, NA1 and ON1 of the RSV-A Taiwan isolates, and wave 3 to wave 5 of the RSV-B Taiwan isolates were involved in the selection-pressure analysis. Putative selection sites that only appeared within the internal node were excluded. The results revealed 4 putative positive selection sites in both RSV-A and RSV-B Taiwan isolates. Residues under positive selection pressure are listed in Table 3.

**Table 3:** The residues that predicted under positive selection pressure.

RSV-A	amino acid substitution	SLAC *p*-value	FEL *p*-value	IFEL *p*-value
	D237A	NS	NS	0.019
	L274P	<0.001	<0.001	0.021
	*E284dup	0.010	NS	NS
	**E284 + 2K E284 + 2G	NS	0.046	NS
	**L284 + 14P	NS	NS	0.026
RSV-B	amino acid substitution	SLAC *p*-value	FEL *p*-value	IFEL *p*-value
	N121G	NS	0.039	NS
	N154K	NS	0.039	0.035
	I255T P255I	NS	0.006	0.040
	T270A T270I	NS	0.031	0.016

*Represent the codon interrupted duplication event itself.

**Indicated that this codon position was located within the duplicated region.

NS = non-significant.

SLAC: single likelihood ancestor counting, FEL: fixed effects likelihood, IFEL: internal fixed effects likelihood.

## Discussion

Samples involved in this study were randomly picked from viral stocks and the sample size did not reflect to the number of hospitalized patients. The detecting rate of RSV-A and RSV-B represent their proportion for each years (Table 1). The result suggested that RSV-A was the predominant subgroup circulating in northern Taiwan during the study period. All samples from the years 2012–2014 were double tested from the first step: retrieving of frozen supernatant from viral stock and still have unordinary (more than 25%) untypable rates. The virus evolves continually and the false negative rate of PCR genotyping is inevitably growing by time. Since all samples involved in this study were recruited from the RSV positive primary cultures, the untypable samples represented false negative rate of PCR typing (Table 1). For the 52 putative RSV-A and RSV-B co-infection samples, only 16 of 52 samples were confirmed as either RSV-A or RSV-B by sequencing (1 and 15 samples were confirmed as RSV-A and RSV-B, respectively). The sequencing results of these 16 samples for another genotype showed poor signals suggesting a possible false positive detection of the putative co-infections.

We discovered that GA2, GA5, and GA7 were co-circulating during 2000–2004 and that GA5 was the most abundant strain in Taiwan. These results are similar to a study from Belgium in the same period^13^. After 2005, however, GA5 faced decline in Taiwan and GA2 became the predominant genotype with the GA2 subtype ON1 eventually becoming the exclusive strain after 2013, merely 2 years after its first appearance in Taiwan (Fig. 1). The RSV-A subtype ON1 is trending toward predomination among circulating RSV strains after the genesis of a C-terminal duplication as well as RSV-B genotype BA^6,7,14^. Our ON1 strains were first identified in a sample collected in 2011, of which is also the earliest isolate in Taiwan. An estimation of time to most recent common ancestor (tMRCA) indicated that ON1 evolved from the progenitor GA2 subtype NA1 around 2009–2010 which is much later than the times reported by studies using a fragments of approximately 300-bp^5,6^, but is similar to the time reported by a study that analyzed G gene sequences using a fragments of approximately 600-bp^15^.

According to the RSV-B Bayesian tree, wave 3 to wave 5 likely emerged as a consequence of the survival of a single strain from a previous wave (Fig. 2). The tMRCA of waves 3, 4, and 5 were dated back to 2001, 2007, and 2012, respectively; the results were exactly match the collection date of clinical isolates from their previous wave. Two exceptions were noted; excep.1 and excep.2 of the RSV-B strain were not observed in these waves (Fig. 2) and both were isolated in 2005. On other hand, one of the NA4 subtype of RSV-A was isolated in 2012, 10 years after the NA4 subtype isolate last appeared in Taiwan. Both observations suggested that minor strains may still be circulating in Taiwan.

The ON1 subtype is believed to have been derived from an NA1 strain through a single duplication event. Phylogenetic evidence supports the classification of majority of ON1 strains as a monophyletic group; however, a set of strains without 72-bp duplication were clustered with some ON1 strains (KX765894, KX765936, and KX765960) on the basis of robust statistical support and represented as an overlap between NA1 and ON1 on the RSV-A phylogenetic tree (Fig. 1) This phenomenon can also be observed in the wave 1 clade of RSV-B. Three reference strains (KU316158, KU316172, and KU316105) with 60-bp duplication are clustered with other non-duplicated wave 1 strains (Fig. 2). In another study, Schobel *et al*. reported the same circumstance in the RSV-A, and attributed the double occurrence of the duplication event. Their hypothesis was based on a later genesis of paralogs group TN1, which included a mixing of both the 72-bp duplicated strains and non-duplicated strains of subtype ON1^16^. The group TN1, however, still shared a common ancestor with the ON1 exclusive group in their results. Furthermore, both RSV-A and RSV-B, the duplicated strains that clustered with non-duplicated strains did not form a monophyletic group in our results (Supplementary Fig. S1). Therefore, we proposed another opinion: RSV requires approximately 10^5^ viral particles for effective infection^17^, indicating that the duplicated strains may somehow co-circulate with normal strains as a minor part of the mixture from a replicative unit of quasispecies before emerging as the consensus sequence and therefore share the same evolutionary history with some non-duplicated strains^18^. The duplicated strain clustering with non-duplicated strains represented an intermediate state before the duplicated strains become predominant and could be considered as the evolutionary relics that failed to fix the duplicated phenotype and therefore distributed at the overlapping part between the successful clade (i.e. ON1 and BA) and their progenitor on the phylogenetic trees. Under this scenario, the secondary occurrence of the duplication event is not essential and the duplicated strains will be contemporaneous and have rare or no progeny. This implied that rare or no sequences will present the same substitution pattern with these duplicated strains especially after years from their collection date and suggested that the occurrence of the duplication event might be close to the collection date of these strains. Therefore we collected and constructed alignments from most recent common ancestor of the overlapping region and nearest successful monophyletic group of duplicated strains from our dataset in chronological order to show the substitution pattern between the overlapping region and nearest duplicated strains (available in Supplementary Dataset). The substation patterns were showed in Supplementary Figs S3 and S4). In the Supplementary Fig. S3, the 72-nt duplicated strains KX765894, KX765936, and KX765960 that clustering with those non-duplicated strains were labeled. KX765936 and KX765960 showed some unique mutations, and shared a few similar mutations with KX765894 that were clustered together with two of our samples (R100-15 and R101-16). Both R100-15 and R101-16 do not have the 72-nt duplication and are not possible a cross contamination with KX765894 (collect from New Zealand), which implied they may share the same common ancestor. By doing BLAST for the analyzed region of this study, the sequences that exactly match to KX765894 were all from the same submission source including itself. No any sequence that exactly matches to KX765936 and KX765960 can be found in GenBank up till date. Within the 72-nt duplication region, both KX765894 and KX765936 were identical to the original version of the duplication region. KX765960 have a C to T substitution that similar to our sample R104-05-GA-TWN-2015 but without any other similar substitution in the rest part. In the Supplementary Fig. S4, the 60-nt duplicated strains KU316158, KU316172, and KU316105, that clustering with those non-duplicated strains present only unique mutations, except the KU316158 have a C to T substitution within in the duplicated region that similar to substitution but without any other similar substitution in the rest part. No other exact matched sequences can be found by BLAST search. Since all the duplicated strains that clustered with non-duplicated strains were obtained from GenBank database rather than our own samples, it is difficult to confirm if these duplicated strains are artifacts in the sequencing assembly process. Nevertheless, our speculation may provide a new insight for future studies.

The population dynamic patterns of RSV-A and RSV-B in Taiwan (Fig. 3) are delayed by approximately 2 years in comparison with those of global RSVs, suggesting that a local epidemic may exhibit different dynamics from those global epidemics^19,20^. The downward trend in the effective population size of RSV-A during 2000–2005 likely signified a loss of diversity because GA5, and both NA2 and NA4 of GA2 became less common or even disappeared from Taiwan. Explaining the increase in the effective population size of RSV-A in 2010 is difficult; however, the decrease that occurred in 2012 may be associated with the complete replacement of all circulating strains by an invading ON1 strain because of the gain of strong fitness conferred by 72-bp duplication and consequently reduced the overall diversity.

After 2 years, the nature of error-prone replication and quasispecies feature of RSV enabled the effective population to recover. Some lineages may have evolved thereafter^18^, thus establishing their population diversity as even greater than that of past lineages. In contrast to RSV-A, the steady phase during 2000–2008 signified the predomination of BA strains that had been conferred with gain of fitness through 60-bp duplication. The sudden decrease in the effective population size of RSV-B in 2009 likely corresponded to the shift of circulating strains from wave 3 to wave 4. Somehow, one strain that survived past wave 3 successfully recovered its effective diversity and became the founding strain of wave 4. The rise in the effective population of RSV-B in 2014 was likely associated with the coexistence of waves 4 and 5.

Four positively selected sites were possibly involved in subtypes NA1 and ON1 of RSV-A, and in waves 3 to 5 of RSV-B strains in Taiwan strains (Table 3). The substitutions of amino acids D237A and L274P in RSV-A caused a loss of N-glycosylation sites. The 237 residue of RSV-A constituted a well-known immune escape hotspot, but did not demonstrate an altered on the binding affinity for antibodies^21^. The E284 + 2K and L284 + 14P were located within the duplicated 72-bp region, and similar substitutions could be found in some reference strains that had been isolated prior to the duplication event (e.g., E262K and L274P in JX015486 and KF826838), suggesting that the duplicated region may have been subjected to the same selection pressure as the original fragment. The E284 + 2G, conversely, is a unique substitution that has only been observed in one strain (MF496482) isolated in 2015 from Taiwan. It is interesting to follow up whether it is a fixed substitution or not. The positively selected site, residues 274 of RSV-A and 255 of RSV-B, were consistent with the observations of a study that included all known strains before 2006^22^; an analysis was conducted before the emergence of the ON1 strain of RSV-A and wave 2 of RSV-B, suggesting that both sites were under strong selection pressure, irrespective of their lineages.

## Conclusion

In the present study, we employed the most recent information, collected over a long period, pertaining to RSV G gene isolates from children in the northern region of Taiwan from 2000 to 2016. By analyzing these samples, we estimated the tMRCA for currently circulating RSVs and the timescale of lineages and genotype replacement. Genetic variability between RSV strains is a signature characteristic that may alter the pathogenicity and fitness of the virus and contribute to its ability to cause repeated infections and outbreaks through immune system evasion.

## Methods

### Patients and samples

RSVs were obtained from virus stocks collected between July 2000 and June 2016 from nasopharyngeal aspirates (NPAs) and throat swabs of children (ages from 0.1–97.7 months) who had been hospitalized with acute respiratory tract infection in pediatric wards. Ethical approval for this study was obtained from the Mackay Memorial Hospital Committee and informed consent was waived (16MMHIS015).

### Viral stocks

Primary isolates were collected by obtaining throat swabs with sterile cotton buds and NPA fluids from all subjects within 48 hours of hospitalization. The specimens were preserved in standard transport media and inoculated on 4 cell lines (MRC-5 from fibroblast of human fetal lungs, Hep-2, A549 from laryngeal carcinoma, and RD cells from rhabdomyosarcoma) as a stock of respiratory viruses. Cultures exhibiting RSV-specific cytopathological effects were confirmed through reactions with immunofluorescent antibodies and immediately stored at −80 °C without any further passage.

### Viral RNA extraction and reverse transcription

The frozen stock viruses were recovered in HEp-2 cells and harvested from cultures when cytopathological effects were observed in 80% of cells. RSV RNA was extracted from 200 µL of a cultured supernatant using High Pure Viral Nucleic Acid Kit (Roche). A viral cDNA synthesis was performed using random primers and the High-Capacity cDNA Reverse Transcription Kit (Applied Biosystems).

### G gene identification

The primers for reverse transcription and PCR were based on published sequences. RSV-A-specific primers G267 and F131^23^, and RSV-B-specific primers BGF and BGR were used for amplification and sequencing^24^. All samples were amplified through two-step RT-PCR by each primer set. Thermocycling was conducted on a GeneAmp PCR system 9700 thermal cycler (Applied Biosystems) that had been programmed as follows: 95 °C for 10 mins for polymerase activation; 40 cycles of 94 °C for 1 min, 60 °C for 1 min, and 72 °C for 1 min, followed by a final extension at 72 °C for 10 min. PCR products were 760 bp for RSV-A and 801 bp for RSV-B. The PCR products were verified by agarose gel electrophoresis. All PCR products with correct size were sequenced using the Sanger method for subsequent phylogenetic analysis

### Phylogenetic analysis

To estimate the evolution rate and reconstruct the demographic history of Taiwan strains of RSVs, 103 RSV-A and 92 RSV-B nucleotide sequences from GenBank and 276 isolates from this study were included in the phylogenetic analysis. DNA sequences were aligned by codon using MEGA5^25^. Overall, 120 and 105 unique patterns were observed on the first codon, 128 and 125 on the second codon, and 153 and 169 on the third codon for the RSV-A and RSV-B, respectively. Phylogenetic trees of the G gene were reconstructed by Bayesian inference with the best-fit GTR + Γ model using the Markov chain Monte Carlo (MCMC) approach, and sequences were partitioned according to the 3 codon positions, through BEAST software package^26^. A Bayesian skyline plot framework was introduced with a relaxed uncorrelated lognormal distribution (UCLD) clock model to estimate the change in effective population size over time. MCMC was performed with 300,000,000 generations and samples were recorded every 30,000 generations for each run and multiple runs were combined with 5% burnin until the effective sample size for each parameter exceeded 200. Bayesian trees were constructed from combined data for maximum clade credibility.

### Selection pressure

Single Likelihood Ancestral Counting (SLAC), Fixed-Effects Likelihood (FEL), and internal FEL (IFEL) methods were used to detect potentially positively selected sites in isolates of 2 recent bottleneck-like events (the occurrence of subtypes NA1 and ON1 in RSV-A; and the waves 4 and 5 emerged from wave 3 and 4 in RSV-B respectively) at a significance level of p < 0.05^27^.

## Supplementary information

Dataset 1

Supplementary Figures
