# Supplementary material for: Persistence and continuous evolution of the human respiratory syncytial virus in northern Taiwan for two decades
Source: Sci Rep. 2019 Mar 18;9:4704. doi: 10.1038/s41598-019-41332-9 (PMC6423049; doi:10.1038/s41598-019-41332-9)
Supplement: Supplementary file 2 — Supplementary Figures [file 41598_2019_41332_MOESM2_ESM.pdf]

Persistence and continuous evolution of the human respiratory syncytial virus in northern Taiwan for two decades

AUTHORS:

Hsin Chi[1,2,3], Kuang-Liang Hsiao[3], Li-Chuan Weng[3], Chang-Pan Liu[1,3,4], Hsin-Fu Liu[3,5,*]

INSTITUTIONS:

[1]Department of Medicine, MacKay Medicine College, New Taipei, Taiwan

[2]Department of Pediatrics, MacKay Children's Hospital and MacKay Memorial Hospital, Taipei, Taiwan

[3]Department of Medical Research, MacKay Memorial Hospital, Taipei, Taiwan

[4]Division of Infectious Diseases, Department of Internal Medicine, MacKay Memorial Hospital, Taipei, Taiwan

[5]Institute of Bioscience and Biotechnology, National Taiwan Ocean University, Keelung, Taiwan

*Corresponding author: Dr. Hsin-Fu Liu

Department of Medical Research, MacKay Memorial Hospital, Min-Sheng Road 45, New Taipei City 25160, Taiwan

Email: hsinfu@mmh.org.tw

Tel: +886-2-2809-4661 ext.2073

Fax: +886-2-2809-4679

Supplementary Fig. S1.
Bayesian trees labeled with reference strains

RSV-A

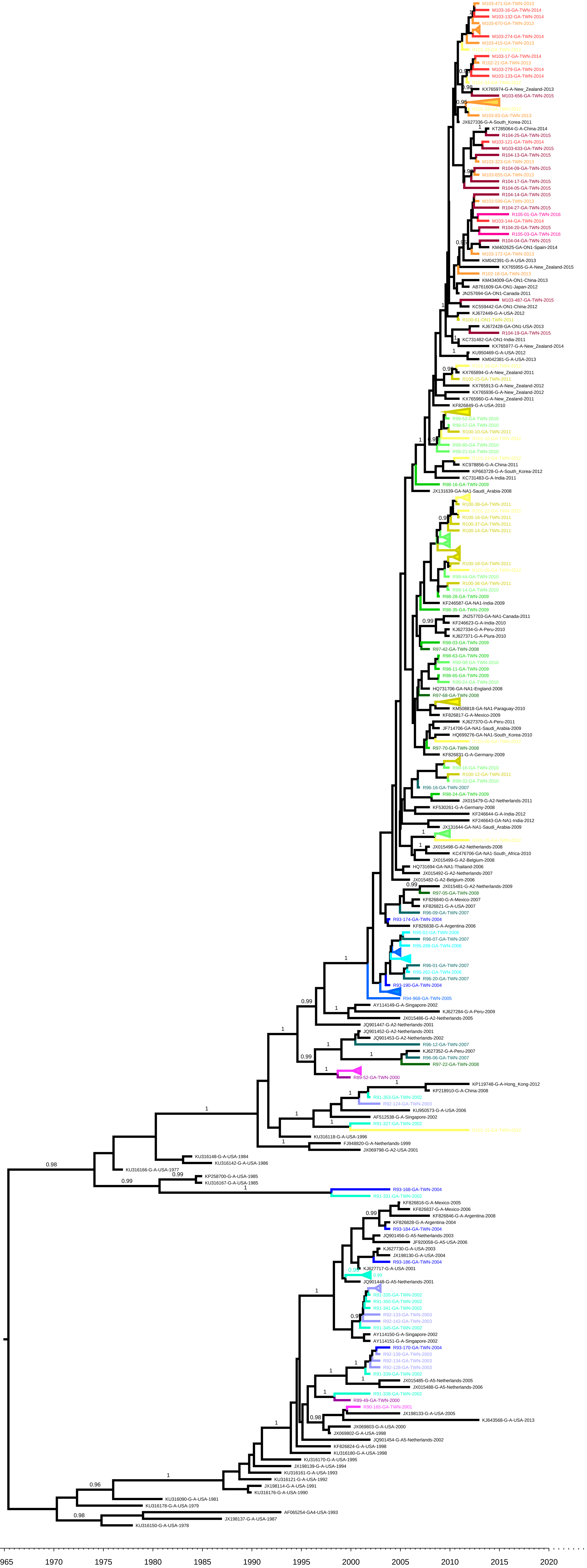

M103-471-GA-TWN-2013
M103-16-GA-TWN-2014
M103-132-GA-TWN-2014
M103-670-GA-TWN-2013
M103-274-GA-TWN-2014
M103-415-GA-TWN-2013
M103-17-GA-TWN-2014
R102-21-GA-TWN-2013
M103-279-GA-TWN-2014
M103-133-GA-TWN-2014
KX765974-G-A-New_Zealand-2013
M103-656-GA-TWN-2015
M103-83-GA-TWN-2013
JX627336-G-A-South_Korea-2011
KT285064-G-A-China-2014
R104-25-GA-TWN-2015
M103-121-GA-TWN-2014
M103-633-GA-TWN-2015
R104-13-GA-TWN-2015
M103-323-GA-TWN-2013
R104-09-GA-TWN-2015
M103-655-GA-TWN-2013
R104-17-GA-TWN-2015
R104-05-GA-TWN-2015
R104-14-GA-TWN-2015
M103-599-GA-TWN-2013
R104-27-GA-TWN-2015
R105-01-GA-TWN-2016
M103-144-GA-TWN-2014
R104-20-GA-TWN-2015
R105-03-GA-TWN-2016
R104-04-GA-TWN-2015
KM402625-GA-ON1-Spain-2014
M103-172-GA-TWN-2013
KM042391-G-A-USA-2013
KX765955-G-A-New_Zealand-2015
R102-18-GA-TWN-2013
KM434009-GA-ON1-China-2013
AB761609-GA-ON1-Japan-2012
JN257694-GA-ON1-Canada-2011
M103-487-GA-TWN-2015
KC559442-GA-ON1-China-2012
KJ672449-G-A-USA-2012
R100-61-ON1-TWN-2011
KJ672428-GA-ON1-USA-2013
R104-19-GA-TWN-2015
KC731482-GA-ON1-India-2011
KX765977-G-A-New_Zealand-2014
KU950469-G-A-USA-2012
KM042381-G-A-USA-2013
KX765894-G-A-New_Zealand-2011
R100-15-GA-TWN-2011
KX765913-G-A-New_Zealand-2012
KX765936-G-A-New_Zealand-2012
KX765960-G-A-New_Zealand-2011
KF826849-G-A-USA-2010
R99-52-GA-TWN-2010
R99-57-GA-TWN-2010
R100-10-GA-TWN-2011
R99-80-GA-TWN-2010
R99-21-GA-TWN-2010
KC978856-G-A-China-2011
KP663728-G-A-South_Korea-2012
KC731483-G-A-India-2011
R98-16-GA-TWN-2009
JX131639-GA-NA1-Saudi_Arabia-2008
R100-38-GA-TWN-2011
R100-16-GA-TWN-2011
R100-37-GA-TWN-2011
R100-14-GA-TWN-2011
R100-18-GA-TWN-2011
R99-44-GA-TWN-2010
R100-36-GA-TWN-2011
R99-14-GA-TWN-2010
R98-28-GA-TWN-2009
KF246587-GA-NA1-India-2009
R98-35-GA-TWN-2009
JN257703-GA-NA1-Canada-2011
KF246623-G-A-India-2010
KJ627334-G-A-Peru-2010
KJ627371-G-A-Piura-2010
R98-03-GA-TWN-2009
R97-42-GA-TWN-2008
R98-63-GA-TWN-2009
R98-08-GA-TWN-2010
R98-11-GA-TWN-2009
R98-65-GA-TWN-2009
R98-24-GA-TWN-2010
HQ731706-GA-NA1-England-2008
R97-68-GA-TWN-2008
KM508818-GA-NA1-Paraguay-2010
KF826820-G-A-Mexico-2009
KJ627370-G-A-Peru-2011
JF714706-GA-NA1-Saudi_Arabia-2009
HQ699276-GA-NA1-South_Korea-2010
R97-70-GA-TWN-2008
KF826831-G-A-Germany-2009
R99-16-GA-TWN-2010
R100-12-GA-TWN-2011
R99-32-GA-TWN-2010
R96-16-GA-TWN-2007
R98-24-GA-TWN-2009
JX015479-G-A2-Netherlands-2011
KF530261-G-A-Germany-2008
KF246644-G-A-India-2012
KF246643-GA-NA1-India-2012
JX131644-GA-NA1-Saudi_Arabia-2009
JX015498-G-A2-Netherlands-2008
KC476706-GA-NA1-South_Africa-2010
JX015499-G-A2-Belgium-2008
HQ731694-GA-NA1-Thailand-2006
JX015492-G-A2-Netherlands-2007
JX015482-G-A2-Belgium-2006
JX015481-G-A2-Netherlands-2009
R97-05-GA-TWN-2008
KF826840-G-A-Mexico-2007
KF826821-G-A-USA-2007
R96-09-GA-TWN-2007
R93-174-GA-TWN-2004
KF826838-G-A-Argentina-2006
R95-02-GA-TWN-2006
R96-07-GA-TWN-2007
R95-288-GA-TWN-2006
R96-01-GA-TWN-2007
R95-262-GA-TWN-2006
R96-20-GA-TWN-2007
R93-190-GA-TWN-2004
R94-968-GA-TWN-2005
AY114149-G-A-Singapore-2002
KJ627284-G-A-Peru-2009
JX015486-G-A2-Netherlands-2005
JQ901447-G-A2-Netherlands-2001
JQ901452-G-A2-Netherlands-2001
JQ901453-G-A2-Netherlands-2002
R96-12-GA-TWN-2007
KJ627352-G-A-Peru-2007
R96-06-GA-TWN-2007
R97-22-GA-TWN-2008
R89-52-GA-TWN-2000
KP119748-G-A-Hong_Kong-2012
KP218910-G-A-China-2008
R91-353-GA-TWN-2002
R92-124-GA-TWN-2003
KU950573-G-A-USA-2006
AF512538-G-A-Singapore-2002
R91-327-GA-TWN-2002
KU316118-G-A-USA-1996
FJ948820-G-A-Netherlands-1999
JX069798-G-A2-USA-2001
KU316148-G-A-USA-1984
KU316142-G-A-USA-1986
KU316166-G-A-USA-1977
KP258700-G-A-USA-1985
KU316167-G-A-USA-1985
R93-168-GA-TWN-2004
R91-331-GA-TWN-2002
KF826816-G-A-Mexico-2005
KF826837-G-A-Mexico-2006
KF826846-G-A-Argentina-2008
KF826828-G-A-Argentina-2004
R93-184-GA-TWN-2004
JQ901456-G-A5-Netherlands-2003
JF920058-G-A5-USA-2006
KJ627730-G-A-USA-2003
JX198130-G-A-USA-2004
R93-186-GA-TWN-2004
KJ627717-G-A-USA-2001
JQ901448-G-A5-Netherlands-2001
R91-335-GA-TWN-2002
R91-350-GA-TWN-2002
R91-341-GA-TWN-2002
R92-133-GA-TWN-2003
R92-142-GA-TWN-2003
R91-345-GA-TWN-2002
AY114150-G-A-Singapore-2002
AY114151-G-A-Singapore-2002
R93-170-GA-TWN-2004
R92-139-GA-TWN-2003
R92-134-GA-TWN-2003
R92-128-GA-TWN-2003
R91-339-GA-TWN-2002
JX015485-G-A5-Netherlands-2005
JX015488-G-A5-Netherlands-2006
R91-338-GA-TWN-2002
R89-49-GA-TWN-2000
R90-165-GA-TWN-2001
JX198133-G-A-USA-2005
KJ643568-G-A-USA-2013
JX069803-G-A-USA-2000
JX069802-G-A-USA-1998
JQ901454-G-A5-Netherlands-2002
KF826824-G-A-USA-1998
KU316180-G-A-USA-1998
KU316170-G-A-USA-1995
JX198139-G-A-USA-1994
KU316161-G-A-USA-1993
KU316121-G-A-USA-1992
JX198114-G-A-USA-1991
KU316176-G-A-USA-1990
KU316090-G-A-USA-1981
KU316178-G-A-USA-1979
AF065254-GA4-USA-1993
JX198137-G-A-USA-1987
KU316150-G-A-USA-1978
0.98
0.99
1
0.96
1965
1970
1975
1980
1985
1990
1995
2000
2005
2010
2015
2020

RSV-B

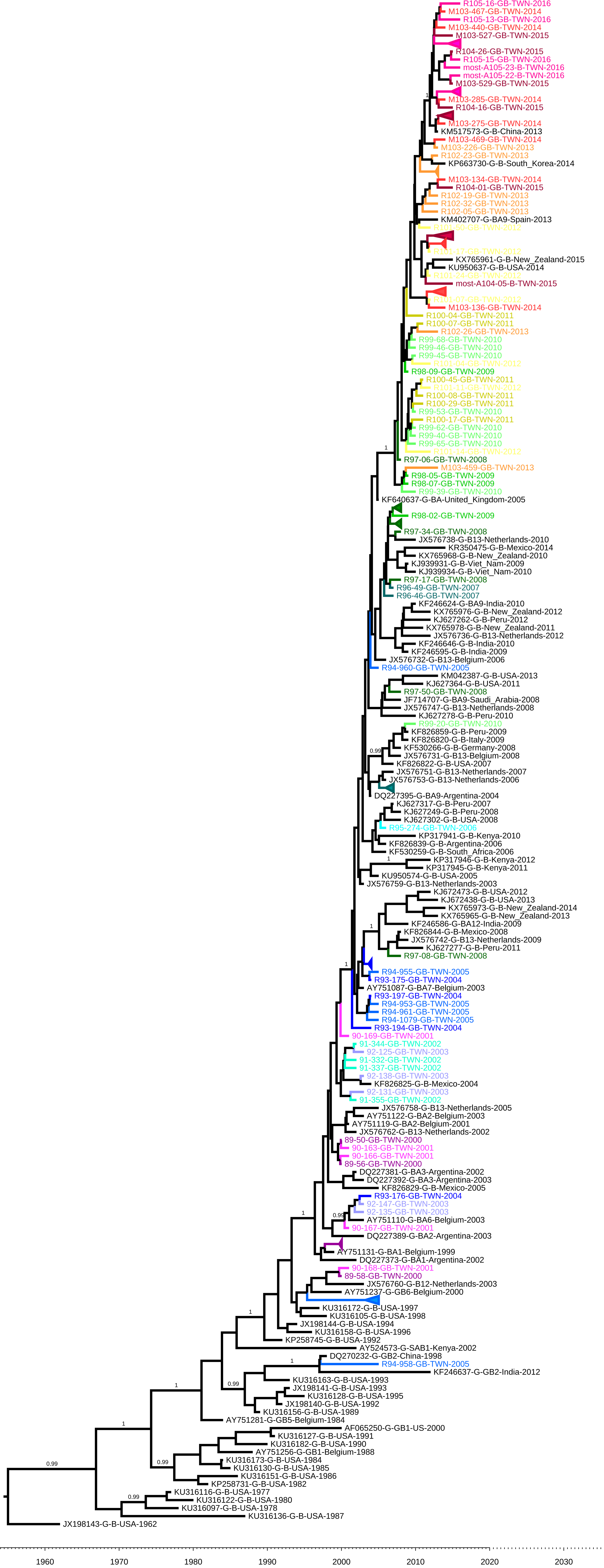

R105-16-GB-TWN-2016
M103-467-GB-TWN-2014
R105-13-GB-TWN-2016
M103-440-GB-TWN-2014
M103-527-GB-TWN-2015
R104-26-GB-TWN-2015
R105-15-GB-TWN-2016
most-A105-23-B-TWN-2016
most-A105-22-B-TWN-2016
M103-529-GB-TWN-2015
M103-285-GB-TWN-2014
R104-16-GB-TWN-2015
M103-275-GB-TWN-2014
KM517573-G-B-China-2013
M103-469-GB-TWN-2014
M103-226-GB-TWN-2013
R102-23-GB-TWN-2013
KP663730-G-B-South_Korea-2014
M103-134-GB-TWN-2014
R104-01-GB-TWN-2015
R102-19-GB-TWN-2013
R102-32-GB-TWN-2013
R102-05-GB-TWN-2013
KM402707-G-BA9-Spain-2013
R101-59-GB-TWN-2012
R101-17-GB-TWN-2012
KX765961-G-B-New_Zealand-2015
KU950637-G-B-USA-2014
R101-24-GB-TWN-2012
most-A104-05-B-TWN-2015
R101-07-GB-TWN-2012
M103-136-GB-TWN-2014
R100-04-GB-TWN-2011
R100-07-GB-TWN-2011
R102-26-GB-TWN-2013
R99-68-GB-TWN-2010
R99-46-GB-TWN-2010
R99-45-GB-TWN-2010
R101-04-GB-TWN-2012
R98-09-GB-TWN-2009
R100-45-GB-TWN-2011
R101-11-GB-TWN-2012
R100-08-GB-TWN-2011
R100-29-GB-TWN-2011
R99-53-GB-TWN-2010
R100-17-GB-TWN-2011
R99-62-GB-TWN-2010
R99-40-GB-TWN-2010
R99-65-GB-TWN-2010
R101-14-GB-TWN-2012
1
R97-06-GB-TWN-2008
M103-459-GB-TWN-2013
R98-05-GB-TWN-2009
R98-07-GB-TWN-2009
R99-39-GB-TWN-2010
KF640637-G-BA-United_Kingdom-2005
R98-02-GB-TWN-2009
R97-34-GB-TWN-2008
JX576738-G-B13-Netherlands-2010
KR350475-G-B-Mexico-2014
KX765968-G-B-New_Zealand-2010
KJ939931-G-B-Viet_Nam-2009
KJ939934-G-B-Viet_Nam-2010
R97-17-GB-TWN-2008
R96-49-GB-TWN-2007
R96-46-GB-TWN-2007
KF246624-G-BA9-India-2010
KX765976-G-B-New_Zealand-2012
KJ627262-G-B-Peru-2012
KX765978-G-B-New_Zealand-2011
JX576736-G-B13-Netherlands-2012
KF246646-G-B-India-2010
KF246595-G-B-India-2009
JX576732-G-B13-Belgium-2006
R94-960-GB-TWN-2005
KM042387-G-B-USA-2013
KJ627364-G-B-USA-2011
R97-50-GB-TWN-2008
JF714707-G-BA9-Saudi_Arabia-2008
JX576747-G-B13-Netherlands-2008
KJ627278-G-B-Peru-2010
R99-20-GB-TWN-2010
KF826859-G-B-Peru-2009
KF826820-G-B-Italy-2009
0.99
KF530266-G-B-Germany-2008
JX576731-G-B13-Belgium-2008
KF826822-G-B-USA-2007
JX576751-G-B13-Netherlands-2007
JX576753-G-B13-Netherlands-2006
DQ227395-G-BA9-Argentina-2004
KJ627317-G-B-Peru-2007
KJ627249-G-B-Peru-2008
KJ627302-G-B-USA-2008
R95-274-GB-TWN-2006
KP317941-G-B-Kenya-2010
KF826839-G-B-Argentina-2006
KF530259-G-B-South_Africa-2006
1
KP317946-G-B-Kenya-2012
KP317945-G-B-Kenya-2011
KU950574-G-B-USA-2005
JX576759-G-B13-Netherlands-2003
KJ672473-G-B-USA-2012
KJ672438-G-B-USA-2013
KX765973-G-B-New_Zealand-2014
KX765965-G-B-New_Zealand-2013
1
KF246586-G-BA12-India-2009
KF826844-G-B-Mexico-2008
JX576742-G-B13-Netherlands-2009
KJ627277-G-B-Peru-2011
R97-08-GB-TWN-2008
1
R94-955-GB-TWN-2005
R93-175-GB-TWN-2004
AY751087-G-BA7-Belgium-2003
R93-197-GB-TWN-2004
R94-953-GB-TWN-2005
R94-961-GB-TWN-2005
R94-1079-GB-TWN-2005
R93-194-GB-TWN-2004
90-169-GB-TWN-2001
91-344-GB-TWN-2002
92-125-GB-TWN-2003
91-332-GB-TWN-2002
91-337-GB-TWN-2002
92-138-GB-TWN-2003
KF826825-G-B-Mexico-2004
92-131-GB-TWN-2003
91-355-GB-TWN-2002
JX576758-G-B13-Netherlands-2005
AY751122-G-BA2-Belgium-2003
AY751119-G-BA2-Belgium-2001
JX576762-G-B13-Netherlands-2002
89-50-GB-TWN-2000
90-163-GB-TWN-2001
90-166-GB-TWN-2001
89-56-GB-TWN-2000
DQ227381-G-BA3-Argentina-2002
DQ227392-G-BA3-Argentina-2003
KF826829-G-B-Mexico-2005
R93-176-GB-TWN-2004
92-147-GB-TWN-2003
92-135-GB-TWN-2003
1
0.99
AY751110-G-BA6-Belgium-2003
90-167-GB-TWN-2001
DQ227389-G-BA2-Argentina-2003
AY751131-G-BA1-Belgium-1999
DQ227373-G-BA1-Argentina-2002
90-168-GB-TWN-2001
89-58-GB-TWN-2000
JX576760-G-B12-Netherlands-2003
AY751237-G-GB6-Belgium-2000
KU316172-G-B-USA-1997
KU316105-G-B-USA-1998
1
JX198144-G-B-USA-1994
KU316158-G-B-USA-1996
KP258745-G-B-USA-1992
AY524573-G-SAB1-Kenya-2002
1
DQ270232-G-GB2-China-1998
R94-958-GB-TWN-2005
KF246637-G-GB2-India-2012
KU316163-G-B-USA-1993
0.99
JX198141-G-B-USA-1993
KU316128-G-B-USA-1995
JX198140-G-B-USA-1992
KU316156-G-B-USA-1989
1
AY751281-G-GB5-Belgium-1984
1
AF065250-G-GB1-US-2000
KU316127-G-B-USA-1991
KU316182-G-B-USA-1990
AY751256-G-GB1-Belgium-1988
0.99
KU316173-G-B-USA-1984
KU316130-G-B-USA-1985
KU316151-G-B-USA-1986
0.99
KP258731-G-B-USA-1982
KU316116-G-B-USA-1977
0.99
KU316122-G-B-USA-1980
KU316097-G-B-USA-1978
KU316136-G-B-USA-1987
JX198143-G-B-USA-1962
1960
1970
1980
1990
2000
2010
2020
2030

Supplementary Fig. S2.
Putative co-infected RSV strains

RSV-A: 2011

Posterior probabilities are labeled on each branch. Branches with a posterior probability equal or greater than 0.95 were considered to be strongly supported. Isolates from the putative co-infection sample of this study were labeled on their isolate name using the color correlating to its collection date (date, color is the same to Figure 1 and 2) and indicated by arrow.

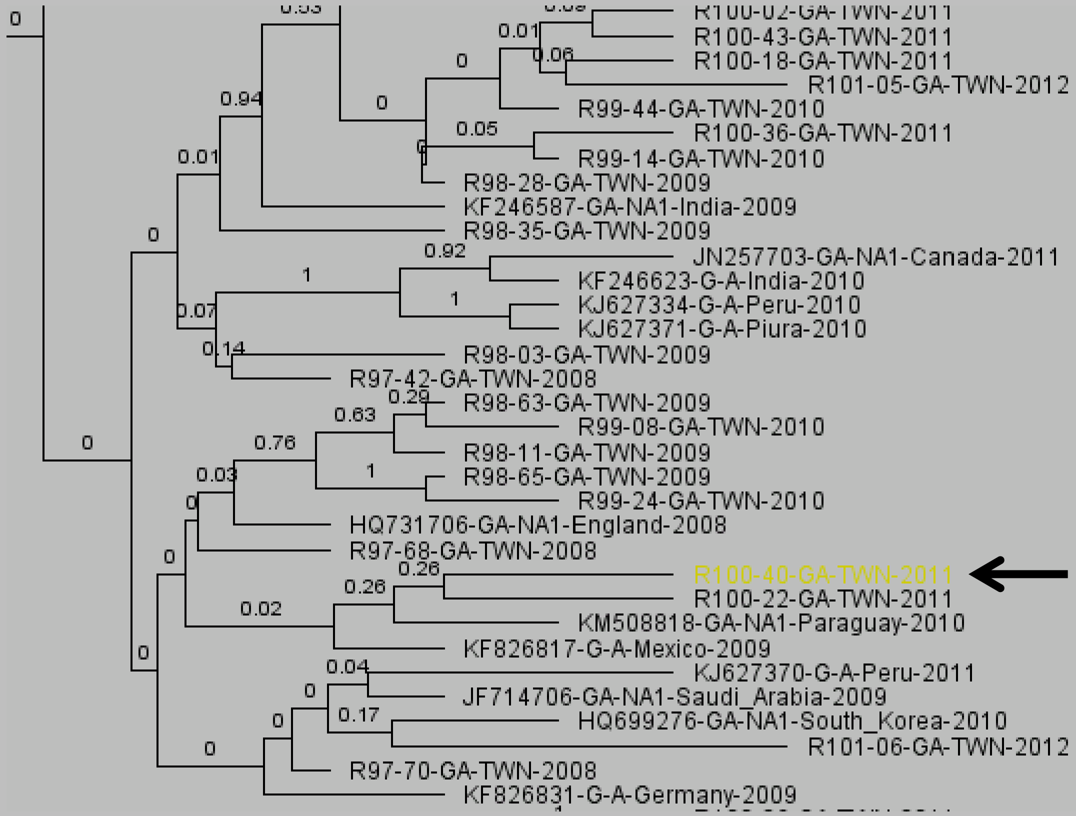

RSV-B: 2000 and 2003

Posterior probabilities are labeled on each branch. Branches with a posterior probability equal or greater than 0.95 were considered to be strongly supported. Isolates from the putative co-infection sample of this study were labeled on their isolate name using the color correlating to its collection date (date, color is the same to Figure 1 and 2) and indicated by arrow.

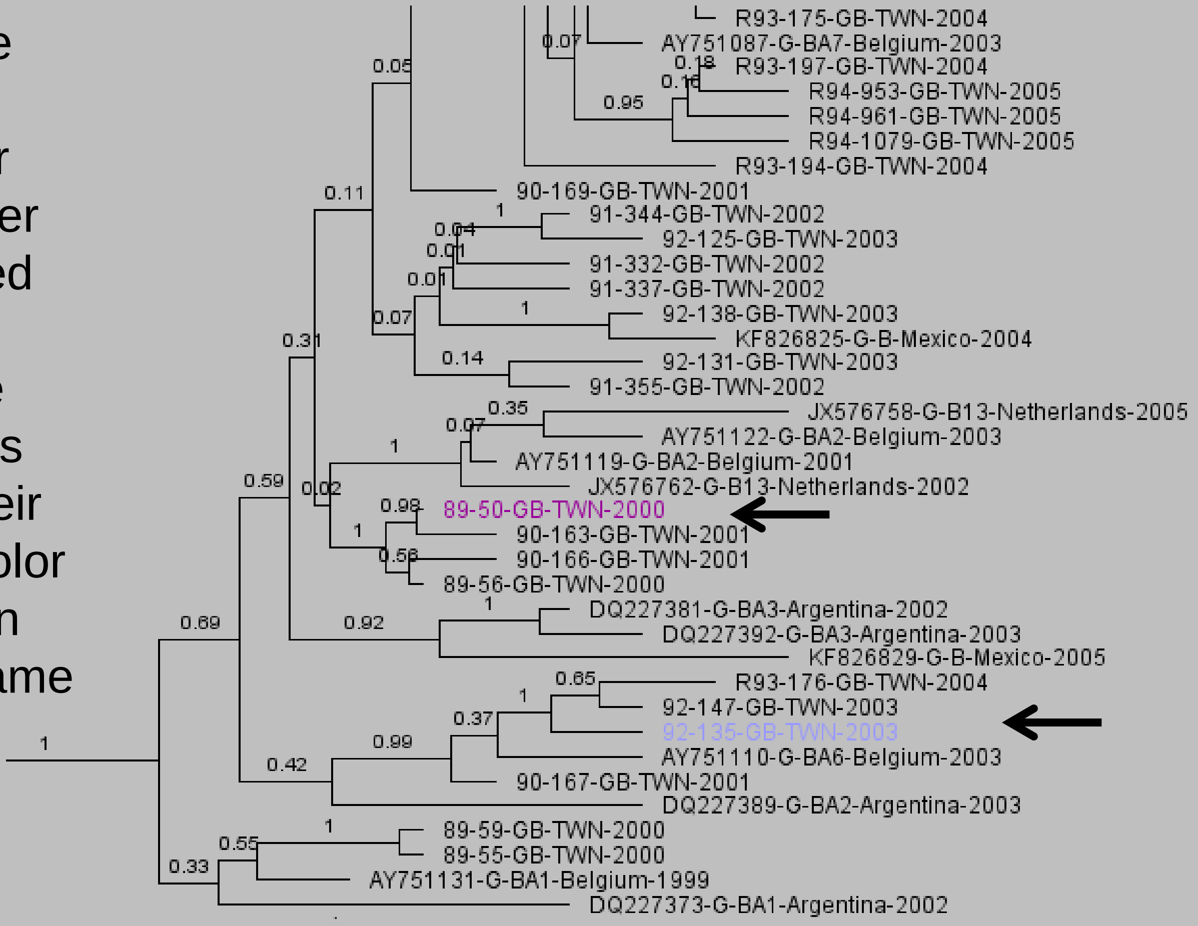

RSV-B: 2008 and 2012

Posterior probabilities are labeled on each branch. Branches with a posterior probability equal or greater than 0.95 were considered to be strongly supported. Isolates from the putative co-infection samples of this study were labeled on their isolate name using colors correlating to their collection date (dates, colors are the same to Figure 1 and 2) and indicated by arrows.

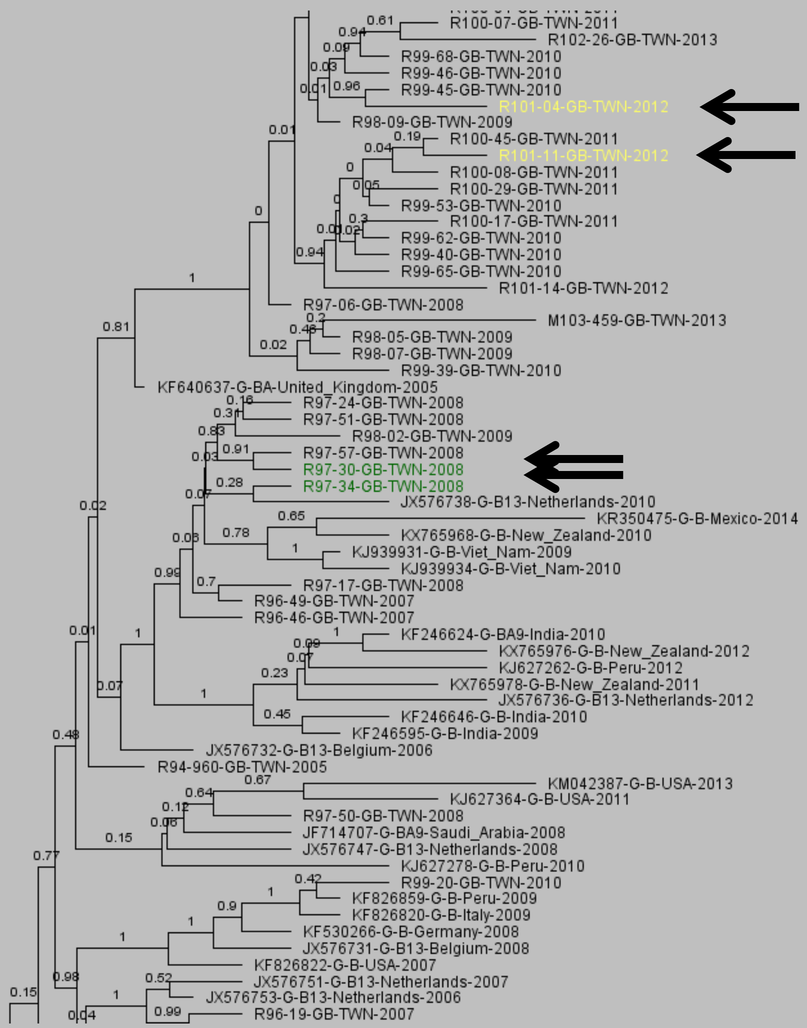

RSV-B: 2014-2016

Posterior probabilities are labeled on each branch. Branches with a posterior probability equal or greater than 0.95 were considered to be strongly supported. Isolates from the putative co-infection samples of this study were labeled on their isolate name using colors correlating to their collection date (dates, colors are the same to Figure 1 and 2) and indicated by arrows.

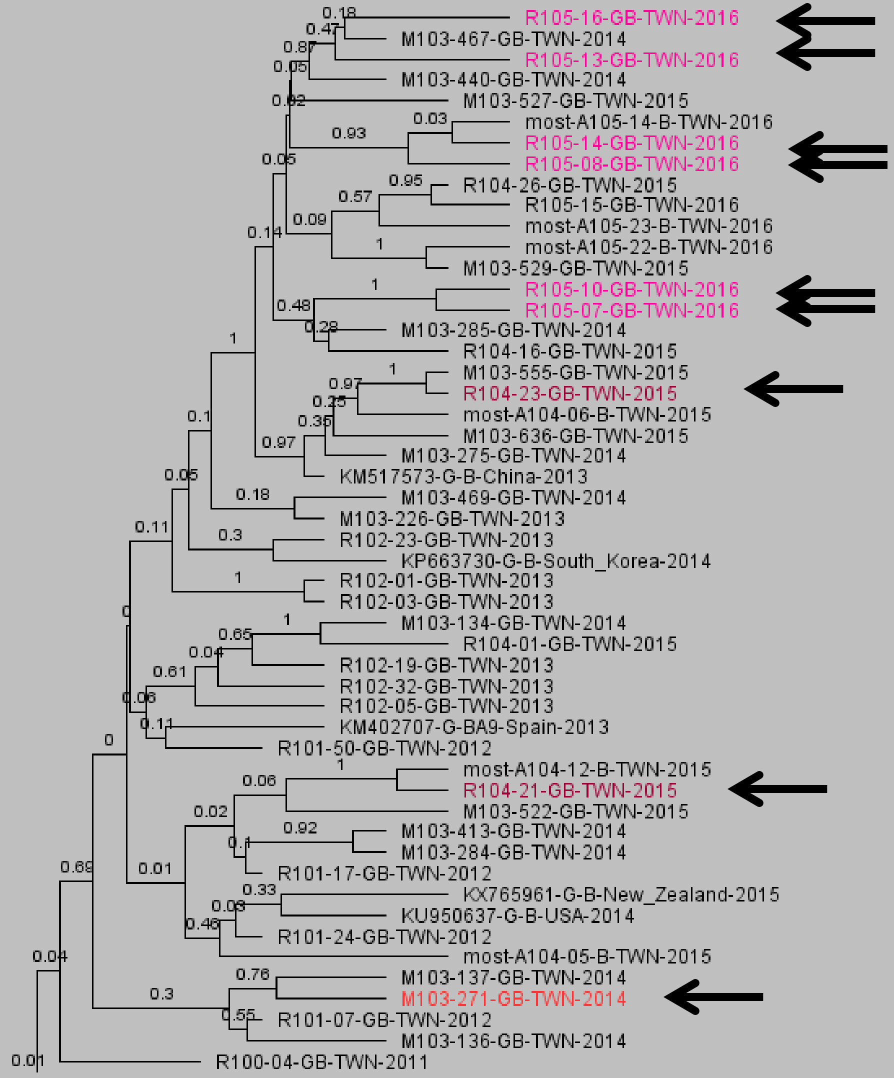

Supplementary Fig. S3.
Alignment of RSV-A overlapping region in chronological order to show substitution pattern

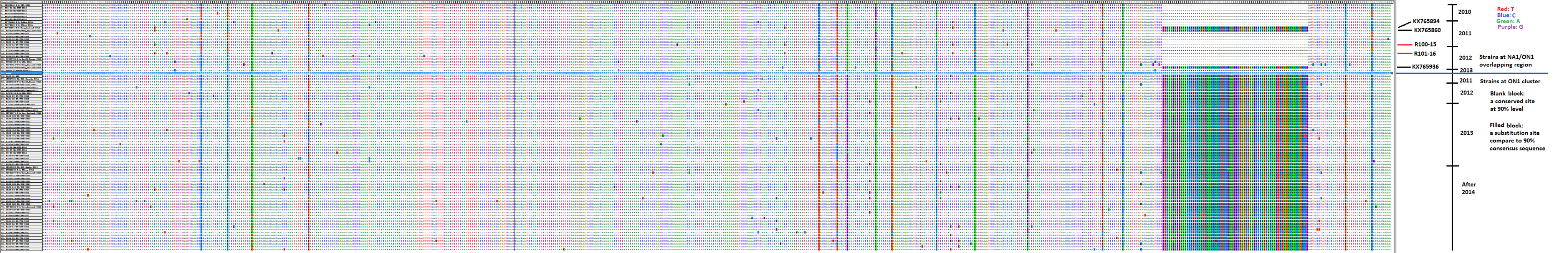

KX765894
KX765860
R100-15
R101-16
KX765936
2010
2011
2012
2013
2011
2012
2013
After 2014
Strains at NA1/ON1 overlapping region
Strains at ON1 cluster
Blank block: a conserved site at 90% level
Filled block: a substitution site compare to 90% consensus sequence
Red: T
Blue: C
Green: A
Purple: G

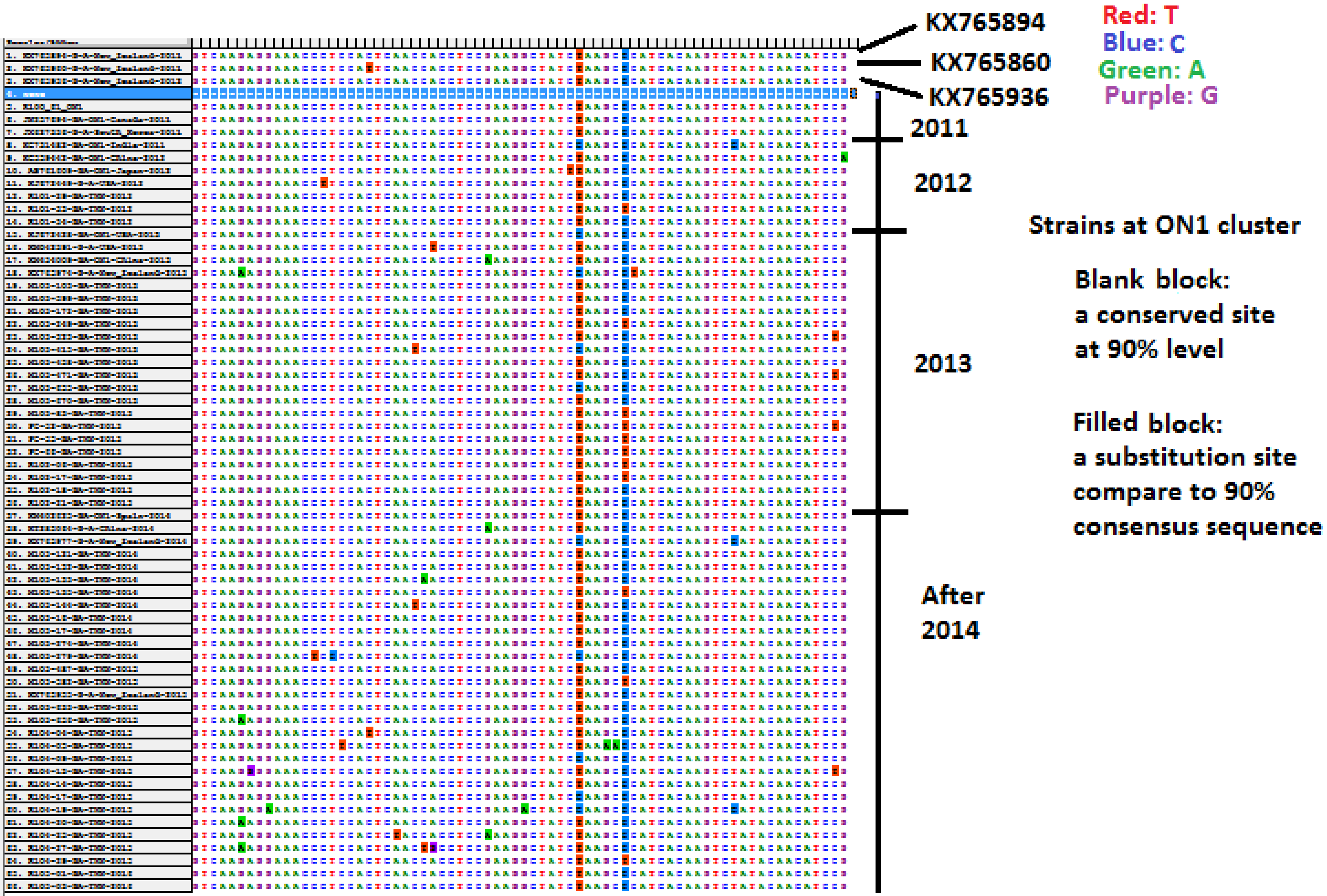
KX765894
KX765860
KX765936
Red: T
Blue: C
Green: A
Purple: G
2011
2012
2013
After 2014
Strains at ON1 cluster
Blank block: a conserved site at 90% level
Filled block: a substitution site compare to 90% consensus sequence

Supplementary Fig. S4.
Alignment of RSV-B overlapping region in chronological order to show substitution pattern

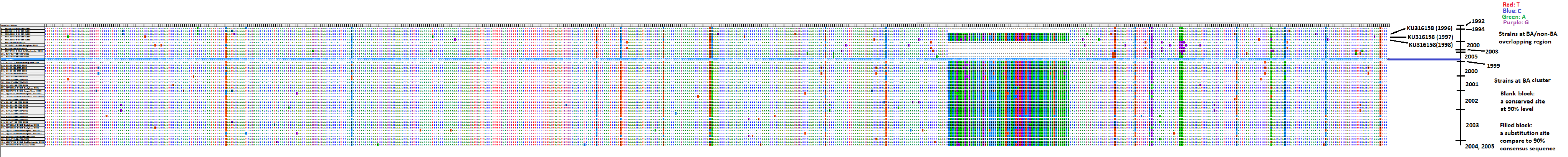

Red: T
Blue: C
Green: A
Purple: G
KU316158 (1996)
KU316158 (1997)
KU316158(1998)
1992
1994
2000
2003
2005
Strains at BA/non-BA overlapping region
1999
2000
2001
2002
2003
2004, 2005
Strains at BA cluster
Blank block: a conserved site at 90% level
Filled block: a substitution site compare to 90% consensus sequence

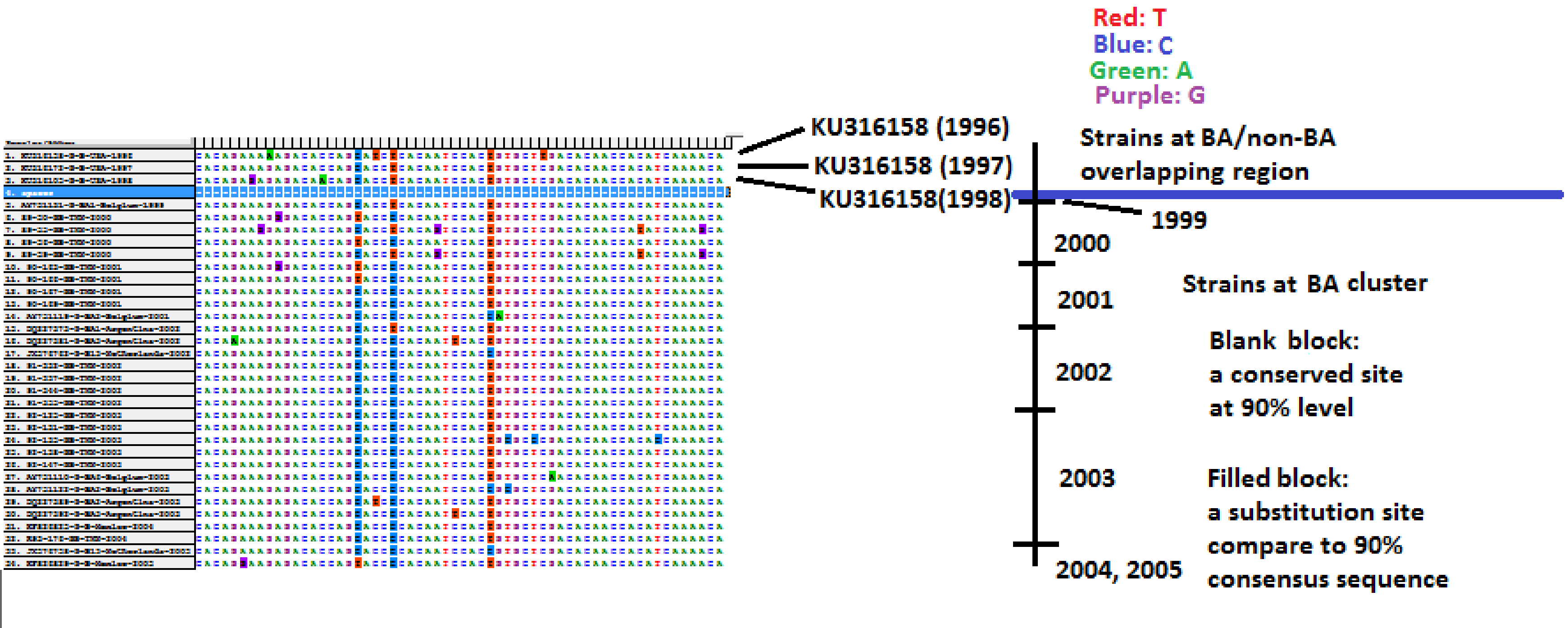
Red: T
Blue: C
Green: A
Purple: G
KU316158 (1996)
KU316158 (1997)
KU316158(1998)
Strains at BA/non-BA
overlapping region
1999
2000
2001
2002
2003
2004, 2005
Strains at BA cluster
Blank block:
a conserved site
at 90% level
Filled block:
a substitution site
compare to 90%
consensus sequence
